# Assessment of wild grapevine (*Vitis vinifera* ssp. *sylvestris*) chlorotypes and accompanying woody species in the Eastern Adriatic region

**DOI:** 10.1371/journal.pone.0199495

**Published:** 2018-06-21

**Authors:** Lukrecija Butorac, Katarina Hančević, Katarina Lukšić, Željko Škvorc, Mario Leko, Erika Maul, Goran Zdunić

**Affiliations:** 1 Institute for Adriatic Crops and Karst Reclamation, Split, Croatia; 2 Faculty of Forestry, University of Zagreb, Zagreb, Croatia; 3 Federal Agromediterranean Institute, Mostar, Bosnia and Herzegovina; 4 Julius Kühn-Institut (JKI), Federal Research Centre for Cultivated Plants, Institute for Grapevine Breeding, Siebeldingen, Germany; National Cheng Kung University, TAIWAN

## Abstract

The Eastern Adriatic region, encompassing Croatia and Bosnia and Herzegovina, is considered an important area of natural populations of wild grapevines (*Vitis vinifera* ssp. *sylvestris*). The wild grapevine arises in the Eastern Adriatic region in a contact zone of the EU-Mediterranean and the sub-Mediterranean characterized by typical karst relief. This study focuses on the chloroplast DNA (cpDNA) analysis of wild grapevines and the biodiversity of accompanying woody species to better understand the genetic variation of the sylvestris populations of the Eastern Adriatic region and to investigate how this variation fits within today’s wild grapevine distribution in the European continent. The allelic variation at nine cpDNA microsatellite loci of wild individuals was used to characterize haplotype diversity in 53 individuals from four population sites. All individuals were grouped into two chlorotypes: A and D, D being the rare haplotype among wild populations on the European continent. In total, 52 woody plant species were identified. However, the studied vegetation structures have been affected by permanent human pressure on natural resources and the preservation status of the collection sites. Based on our results, we conclude that the investigated areas were probably shelter zones for wild grapevine preservation during the unfavorable glaciation era.

## Introduction

Wild grapevines (*Vitis vinifera* ssp. *sylvestris* can be found throughout the northern hemisphere, from the Atlantic coast of Europe and northern Africa to the Western Himalayas. It is believed to be a living ancestor of modern grapevine cultivars [1]. McGovern [2] suggested that humans domesticated wild grapevines for the first time in the upland regions of Eastern Turkey and in northwest Iran between 6000 and 8000 B.C. From the Tertiary to today, the spread of wild grapevine has been influenced by climatic forces, human impact and pathogen dispersal.

Because of anthropogenic impact, natural events, such as floods and fire, and the arrival of mildew diseases and phylloxera in Europe, *V*. *vinifera* ssp. *sylvestris* is a severely threatened species [3–4]. In addition, moderate to high gene flow is possible between wild and cultivated grapevines [5–6]. Moreover, only a negligible part of European forest cover is present in its primary form of virgin forest, the natural habitat of wild grapevines. Over the last two decades, the interest in wild grapevines has increased considerably. As a consequence, in several European countries, *V*. *vinifera* ssp. *sylvestris* surveys have been funded in recent years. As a result of these initiatives, wild grape populations have been discovered in forests and isolated areas not previously surveyed [7–9].

This hydrophilic species is mostly found along riverbanks in the wilderness. As they cannot reproduce in the shade, wild grapevines are occasionally found along roadsides and forest edges growing up on and among trees.

A number of written records testify that the Croatian karst area was once covered with virgin forests [10–13]. However, continuous anthropogenic pressure has resulted in changes in the tree layer canopy, causing biodiversity impoverishment, degradation or even forest disappearance. The early settlement of the Mediterranean resulted in forest degradation [14]. However, fortunately, the geomorphological diversity of forms in this area remains visible because the harsh karstic relief contributed to the preservation of virgin forest cover and other flora in detached microlocations. Shaped by intensive action of surface and underground water as well as high temperature differences in the respective seasons, karstic relief abounds with forms such as cracks, grooves, rocky areas, pits and caves. The dominant soil of this area is calcocambisol formed on limestone and dolomite as parent materials [14]. Additionally, the wild grapevine plant exhibits high tolerance to soil lime [15–16]. Therefore, areas of karstic relief represent potential refuges for wild grapevine.

“Reading”the grapevine genome at the molecular level presents a good opportunity to clarify several major issues, such as the relationships between the two subspecies; wild and cultivated grapevine (*Vitis vinifera* ssp. *sativa*), parentage, domestication events and the history of wild grapevine migration as well as how to find resistance genes for breeding purposes [17–19]. Plastid DNA, such as chloroplast DNA (cpDNA), in most Angiosperms displays a variable number of mononucleotide repeats and provides an opportunity to analyze the genetic structure of a population and to address phylogeographical issues in plant species [20–24]. Since the cpDNA germplasm is maternally transmitted to the offspring, phylogeny derivation from chloroplast genome data with a low mutation rate and no recombination events is an excellent complement to the standard nuclear DNA genetic approach to reconstructing evolutionary relationships.

Information based on non-recombinant and uniparental inherited cpDNA as well as the population structure of accompanying plant woody species invites us to go back in time and investigate the routes, bridges and spatial isolation that influenced the present state of wild grapevine distribution in Eastern Europe. Although information on the cpDNA profiling of wild samples collected from the Middle East to Western Europe is available, an information gap remains regarding wild grapevines in the Balkan Peninsula between Turkey and Italy, where Croatia and Bosnia and Herzegovina are located. To obtain new information on wild grapevine genetic resources and the diversity of accompanying woody plant associations in the mentioned areas, this research was designed to describe the natural level of haplotype variability. How wild grapevines and their accompanying woody species in Croatia and Bosnia and Herzegovina contribute to the global picture of grapevine resources, evolution and phylogeography will be discussed in this paper.

## Materials and methods

### Collection sites

Four populations of wild grapevines previously genotyped by SSR markers [9] and the associated woody species from the terrains distinctive for wild grape habitats were included in the analysis. The collection sites were in the Paklenica, Neretva, Imotski, and Gizdavac regions. The permission for scientific sampling in National Park Paklenica was issued by The Ministry of Environment and Energy, Republic of Croatia. For other locations no specific permission was required. Wild grapevines and associated woody species were sampled over a surface area of 400 m^2^ per location. The main characteristics of the sites are provided in Table 1. For average annual precipitation (AP) and temperature (AT) we used climate data from the WorldClim database [25].

**Table 1:** Collection site characteristics and number of genotyped individuals.

Location	Altitude (m)	Coordinates (longitude, latitude)	Average annual precipitation (mm)	Average annual temperature (°C)	Number of analyzed individuals
**Paklenica**	198	44°18.232' N, 15°28.288' E	1109	11.8	19
**Neretva**	133	43°33.034' N, 17°43.353' E	1297	13.7	20
**Imotski**	313	43°26.963' N, 17°12.510' E	1117	13.1	11
**Gizdavac**	377	43°39.334' N, 16°29.302' E	950	13.4	3

Each location exhibits a different stage of flora preservation.

Paklenica: no signs of logging or other human intervention in the research area was observed. Based on historical archival evidence, the region was not disturbed, and the entire forest has been under strict protection since it was declared a National Park in 1949.

Neretva: the largest karstic river in the Dinaric Alps in the Eastern part of the Adriatic basin/watershed. The majority of this area exhibits different levels of national and local ecological protection. The sampling was conducted in the area of minimal or no protection.

Imotski: the samples were collected at Modro jezero or Blue Lake, a karstic lake located in the vicinity of urban centers. The lake occupies a deep sinkhole, and the appearance of the lake is attributed to the collapse of underground water caves in a region that suffers from occasional earthquakes.

Gizdavac: the most devastated location compared to others investigated here. The vicinity of urban centers has influenced the location’s present state, which can be defined as degraded pubescent oak forest.

### Genetic analysis based on chloroplast SSR markers

A set of samples, in total 53 wild individuals (Table 1), was used in the cpDNA analysis. The plant sampling strategy was the same for all samples and populations. Young, healthy leaves were collected at the four collection sites and placed in plastic bags filled with hygroscopic silica gel beads (Sigma-Aldrich) to dry the leaves until further laboratory processing.

### DNA extraction and microsatellite analysis

Total DNA was extracted using a NucleoSpin Plant II kit (Macherey-Nagel, Düren, Germany). The extracted DNA was quantified and used as a working DNA concentration of 1 ng/μL. A set of 9 microsatellite loci were analyzed to study the genetic diversity of cpDNA: cpSSR3, cpSSR5, cpSSR10, NTCP8, NTCP12, ccSSR5, ccSSR9, ccSSR14, ccSSR23 [21, 26].

The KAPA Fast Multiplex PCR Kit (2x) (KapaBiosystems, USA) was used to create reaction mixtures containing the master mix, 100 pmol of each primer and approximately 1 ng of template DNA. Amplification was performed in ABI 9700 thermal cyclers (Applied Biosystems) using the following program: 15 min initial denaturation at 95°C, followed by 30 cycles of denaturation at 94°C (30 s), annealing at 60°C (90 s) and extension at 72°C (60 s). A final extension was performed at 60°C for 40 min.

The amplified products were resolved using capillary electrophoresis on an ABI 3130xl Genetic Analyzer (Applied Biosystems, Foster City, California, USA) using GeneScan-LIZ 500 as an internal standard. Peaks were identified by size and height with GeneMapper 5.0 software (Applied Biosystems).

### Analysis of woody plant species

At the collection sites, all woody species (i.e., shrubs and trees) were collected to assess the forest vegetation that supports wild grapevine growth and to estimate the (abiotic) conditions at the sites. Herbaceous plants, mosses and ferns were not considered. All species were brought to the laboratory for definitive identification and a herbarium encompassing all the collected plants was made. The names of the woody species follow the Euro+Med PlantBase [27].

For each location average Ellenberg indicator value for light, temperature, moisture, soil reaction and soil nutrients availability was calculated. Average Ellenberg indicator values [28–29] were used to indirectly characterize the environmental factors: light (L), temperature (T), moisture (F), soil reaction (R) and soil nutrient availability (N). The values were described to summarize interactions between plants and environment while recognizing the role of each species as a biological indicator [28–30]. The calculation of the average Ellenberg values for each site was performed for all woody species present at the site. Continentality indicator value (K) was excluded from the analysis, due to the small gradient value.

### Data analysis

Different measures of genetic variability among 53 unique genotypes at 9 cpSSR loci were calculated. The number of effective alleles (Ne), Shannon's information index (I), and genetic diversity (h) were calculated for each locus using GenAlEx 6.5 [31]. Haplotype frequency was measured as a percentage of individuals sharing the same haplotype in the population and the collection samples in general.

The floristic composition was elaborated using Non-Metric Multidimensional Scaling (NMDS) and Bray-Curtis dissimilarity measure. Twenty iterations were used. The Ellenberg indicator values were passively projected as vectors plotted on the bi-dimensional space on the NMDS plot. Analysis was performed in R [32] using the vegan package [33].

## Results

Fifty-three unique individuals of wild grapevine were analyzed with cpDNA microsatellite markers. DNA amplifications with the 9 primer pairs used for the chloroplast SSR analysis revealed that 8 analyzed loci were polymorphic (Table 2; cpSSR3, cpSSR5, cpSSR10, NTCP12, ccSSR5, ccSSR9, ccSSR14, ccSSR23). Only the allele NTCP8 exhibited no polymorphism in the studied accessions. A total of 16 different allele variants were found in the remaining 8 microsatellite loci. The number of effective alleles was the lowest in cpSSR10 (1.534), followed by ccSSR9 and ccSSR23 (1.550) while the other loci had the same value (1.539). Shannon’s informational index varied between 0.533 and 0.540 confirming that all tested loci except NTCP8 were distinct (Table 2). Shannon’s informational index was the highest with ccSSR9 and ccSSR23 (0.540).

**Table 2:** Allele sizes (bp), allele frequencies, number of effective alleles (Ne), Shannon's information index (I), and genetic diversity (h) for nine chloroplasic SSR loci in the 53 Adriatic wild individuals.

Locus	Allele size	Allele frequency	Ne	I	h
**cpSSR3**	**106**	0.774	1.539	0.535	0.350
	**107**	0.226			
**cpSSR5**	**104**	0.226	1.539	0.535	0.350
	**105**	0.774			
**cpSSR10**	**114**	0.776	1.534	0.533	0.348
	**115**	0.224			
**NTCP8**	**248**	1.000	1.000	0.000	0.000
**NTCP12**	**118**	0.226	1.539	0.535	0.350
	**119**	0.774			
**ccSSR5**	**254**	0.226	1.539	0.535	0.350
	**255**	0.774			
**ccSSR9**	**165**	0.231	1.550	0.540	0.355
	**166**	0.769			
**ccSSR14**	**201**	0.774	1.539	0.535	0.350
	**202**	0.226			
**ccSSR23**	**280**	0.769	1.550	0.540	0.355
	**281**	0.231			

Table 3 shows that the size variants of 8 loci combine in two different haplotypes: A and D. Haplotype A was found with a frequency of 77.8% in the processed samples and haplotype D in 22.4%. However, the haplotype frequency oscillates among the populations. Wild grapevines from Neretva, Paklenica and Gizdavac sites were all mixed populations with a prevalence of the A haplotype in 95% in Neretva and 89.5% in Paklenica samples analyzed. Although the majority of haplotype A was also found in Gizdavac collection site, no prevalence of haplotypes in percentages for Gizdavac location was given regarding to only three individuals presented at that location. In the Imotski population, haplotype D was predominant and found in 80% of samples (Table 3).

**Table 3:** Size variance of 9 microsatellite chloroplast loci and associated chlorotypes of 53 wild individuals from the four collection sites.

Sample name and Country of origin	cpSSR3	cpSSR5	cpSSR10	NTCP8	NTCP12	ccSSR5	ccSSR9	ccSSR14	ccSSR23	Chlorotype
GZ1 (CRO)	106	105	N/A	248	119	255	166	201	280	A
GZ2 (CRO)	107	104	N/A	248	118	254	165	202	281	D
GZ3 (CRO)	106	105	114	248	119	255	166	201	280	A
IM1 (CRO)	107	104	115	248	118	254	165	202	281	D
IM2 (CRO)	107	104	115	248	118	254	165	202	281	D
IM3 (CRO)	107	104	115	248	118	254	165	202	281	D
IM4 (CRO)	107	104	115	248	118	254	165	202	281	D
IM5 (CRO)	107	104	115	248	118	254	165	202	281	D
IM6 (CRO)	107	104	115	248	118	254	165	202	281	D
IM7 (CRO)	106	105	114	248	119	255	166	201	280	A
IM8 (CRO)	107	104	115	248	118	254	165	202	281	D
IM9 (CRO)	106	105	114	248	119	255	166	201	280	A
IM10 (CRO)	107	104	115	248	118	254	165	202	281	D
IM11 (CRO)	106	105	114	248	119	255	166	201	280	A
NE1 (BIH)	106	105	114	248	119	255	166	201	280	A
NE2 (BIH)	106	105	114	248	119	255	166	201	280	A
NE3 (BIH)	106	105	114	248	119	255	166	201	280	A
NE4 (BIH)	106	105	114	248	119	255	166	201	280	A
NE5 (BIH)	106	105	114	248	119	255	166	201	280	A
NE6 (BIH)	107	104	115	248	118	254	165	202	281	D
NE7 (BIH)	106	105	114	248	119	255	166	201	280	A
NE8 (BIH)	106	105	114	248	119	255	166	201	280	A
NE9 (BIH)	106	105	114	248	119	255	166	201	280	A
NE10 (BIH)	106	105	N/A	248	119	255	N/A	201	N/A	A
NE11 (BIH)	106	105	114	248	119	255	166	201	280	A
NE12 (BIH)	106	105	114	248	119	255	166	201	280	A
NE13 (BIH)	106	105	114	248	119	255	166	201	280	A
NE14 (BIH)	106	105	114	248	119	255	166	201	280	A
NE15 (BIH)	106	105	114	248	119	255	166	201	280	A
NE16 (BIH)	106	105	114	248	119	255	166	201	280	A
NE17 (BIH)	106	105	114	248	119	255	166	201	280	A
NE18 (BIH)	106	105	114	248	119	255	166	201	280	A
NE19 (BIH)	106	105	114	248	119	255	166	201	280	A
NE20 (BIH)	106	105	114	248	119	255	166	201	280	A
PK1 (CRO)	106	105	114	248	119	255	166	201	280	A
PK2 (CRO)	107	104	115	248	118	254	165	202	281	D
PK3 (CRO)	106	105	114	248	119	255	166	201	280	A
PK4 (CRO)	106	105	114	248	119	255	166	201	280	A
PK5 (CRO)	106	105	114	248	119	255	166	201	280	A
PK6 (CRO)	106	105	114	248	119	255	166	201	280	A
PK7 (CRO)	106	105	114	248	119	255	166	201	280	A
PK8 (CRO)	106	105	114	248	119	255	166	201	280	A
PK9 (CRO)	106	105	114	248	119	255	166	201	280	A
PK10 (CRO)	107	104	115	248	118	254	165	202	281	D
PK11 (CRO)	106	105	N/A	248	119	255	166	201	280	A
PK12 (CRO)	106	105	114	248	119	255	166	201	280	A
PK13 (CRO)	106	105	114	248	119	255	166	201	280	A
PK14 (CRO)	106	105	114	248	119	255	166	201	280	A
PK15 (CRO)	106	105	114	248	119	255	166	201	280	A
PK16 (CRO)	106	105	114	248	119	255	166	201	280	A
PK17 (CRO)	106	105	114	248	119	255	166	201	280	A
PK18 (CRO)	106	105	114	248	119	255	166	201	280	A
PK19 (CRO)	106	105	114	248	119	255	166	201	280	A

N/A–not amplified.

At all four collection sites, a total of 52 woody species, i.e., trees and shrubs, were identified. The largest variability of species was found in the Neretva study area: 39 (Table 4). Thirty-one supporter plants were found in Paklenica, 16 in Gizdavac and only 8 in Imotski (Table 4).

**Table 4:** Botanical supporters of wild grapevine found at the four location sites.

Location	Botanical supporters
**Paklenica**	*Acer campestre*, *Acer monspessulanum*, *Acer pseudoplatanus*, *Asparagus acutifolius*, *Calluna vulgaris*, *Carpinus orientalis*, *Celtis australis*, *Clematis vitalba*, *Colutea arborescens*, *Cornus mas*, *Coronilla emerus*, *Crataegus monogyna*, *Fagus sylvatica*, *Ficus carica*, *Fraxinus ornus*, *Hedera helix*, *Juniperus communis*, *Juniperus oxycedrus*, *Morus alba*, *Ostrya carpininifolia*, *Paliurus spina-christi*, *Phillyrea latifolia*, *Pinus halepensis*, *Pinus nigra*, *Pistacia lentiscus*, *Pistacia terebinthus*, *Prunus mahaleb*, *Rosa canina*, *Rubus sp*., *Salvia officinalis*, *Sambucus nigra*
**Neretva**	*A*. *monspessulanum*, *Ailanthus altissima*, *Arbutus unedo*, *A*. *acutifolius*, *C*. *vulgaris*, *C*. *orientalis*, *C*. *australis*, *C*. *vitalba*, *C*. *arborescens*, *C*. *mas*, *Cotinus coggygria*, *C*. *monogyna*, *F*. *carica*, *Frangula alnus*, *Fraxinus angustifolia*, *Fraxinus excelsior*, *F*. *ornus*, *H*. *helix*, *Ilex aquifolium*, *Juglans regia*, *J*. *oxycedrus*, *Lonicera caprifolium*, *Morus nigra*, *P*. *spina-christi*, *Petteria ramentacea*, *P*. *latifolia*, *P*. *nigra*, *P*. *lentiscus*, *P*. *mahaleb*, *Punica granatum*, *Quercus cerris*, *Quercus ilex*, *Quercus pubescens*, *Rhamnus alaternus*, *Robinia pseudoacacia*, *R*. *canina*, *Rubus sp*., *Thymus serpyllum*, *Viburnum tinus*
**Imotski**	*A*. *acutifolius*, *C*. *vulgaris*, *C*. *vitalba*, *Helichrysum italicum*, *P*. *spina-christi*, *P*. *ramentacea*, *P*. *mahaleb*, *Rubus sp*.
**Gizdavac**	*A*. *monspessulanum*, *A*. *acutifolius*, *C*. *orientalis*, *C*. *australis*, *C*. *arborescens*, *C*. *emerus*, *C*. *monogyna*, *F*. *ornus*, *J*. *communis*, *P*. *spina-christi*, *P*. *mahaleb*, *P*. *granatum*, *Q*. *pubescens*, *R*. *alaternus*, *Rubus sp*., *Satureja montana*

**Fig 1:**
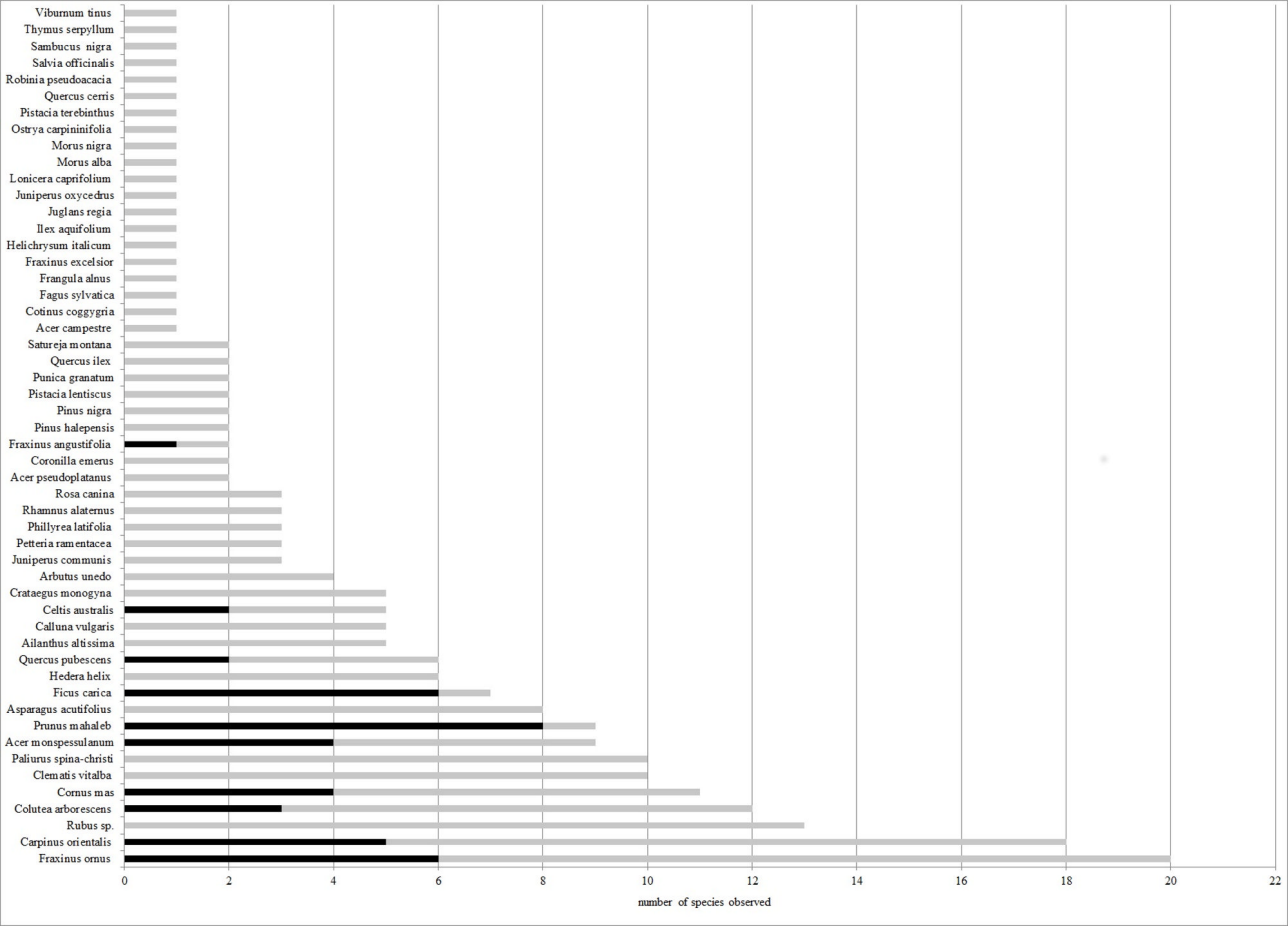
Accompanying woody species and plants supporting climbing wild grapevines. Showing number of trees and shrubs observed on 400 m^2^ in gray column and number of plants supporting climbing wild grapevines in black.

The main supporters of wild grapevine populations were typical woody species from Mediterranean karst forests (Fig 1).

As shown in Fig 1, the most commonly found species of accompanying flora are as follows: *Fraxinus ornus*, *Carpinus orientalis*, *Rubus sp*., *Colutea arborescens* and *Cornus mas*with 20, 18, 13, 12 and 11 occurrences, respectively. The following species were the most commonly supporting plants for grapevine climbing lianas: *Prunus mahaleb*, *Ficus carica*, *F*. *ornus* and *C*. *orientalis* with 8, 6, 6 and 5 occurrences, respectively.

Components of the climax vegetation, such as *Quercus ilex* and *Q*. *pubescens*, appeared a short distance from the wild grapevine populations. In addition to the preceding two species, two basic conifer species also occurred in these areas: *Pinus halepensis* and *P*. *nigra*. The forests contained a number of deciduous tree species that accompany *Quercus*: *Acer monspessulanum*, *A*. *campestre*, *Fraxinus ornus*, *Carpinus orientalis*. In the higher parts of Paklenica, *Fagus sylvatica* was present but only in a small part of the area.

The shrubs most commonly found as accompanying flora were: *Arbutus unedo*, *Asparagus acutifolius*, *Colutea arborescens*, *Cornus mas*, *Crataegus monogyna*, *Paliurus spina-christi*, *Phillyrea latifolia*, *Rubus sp* and *Viburnum tinus*. Climbing species included *Clematis vitalba* and *Hedera helix* in addition to wild grapevines. *Ficus carica* and *Prunus mahaleb* were observed relatively frequently (Fig 1). Relatively recently introduced invasive species, such as *Ailanthus altissima*, were also found (Fig 1).

As a result of edaphic conditions in the Imotski location, only eight different woody species were identified: *Asparagus acutifolius*, *Calluna vulgaris*, *Clematis vitalba*, *Hedera helix*, *Paliurus spina-christi*, *Petteria ramentacea*, *Prunus mahaleb*, *Rubus sp*. (Table 4).

**Fig 2:**
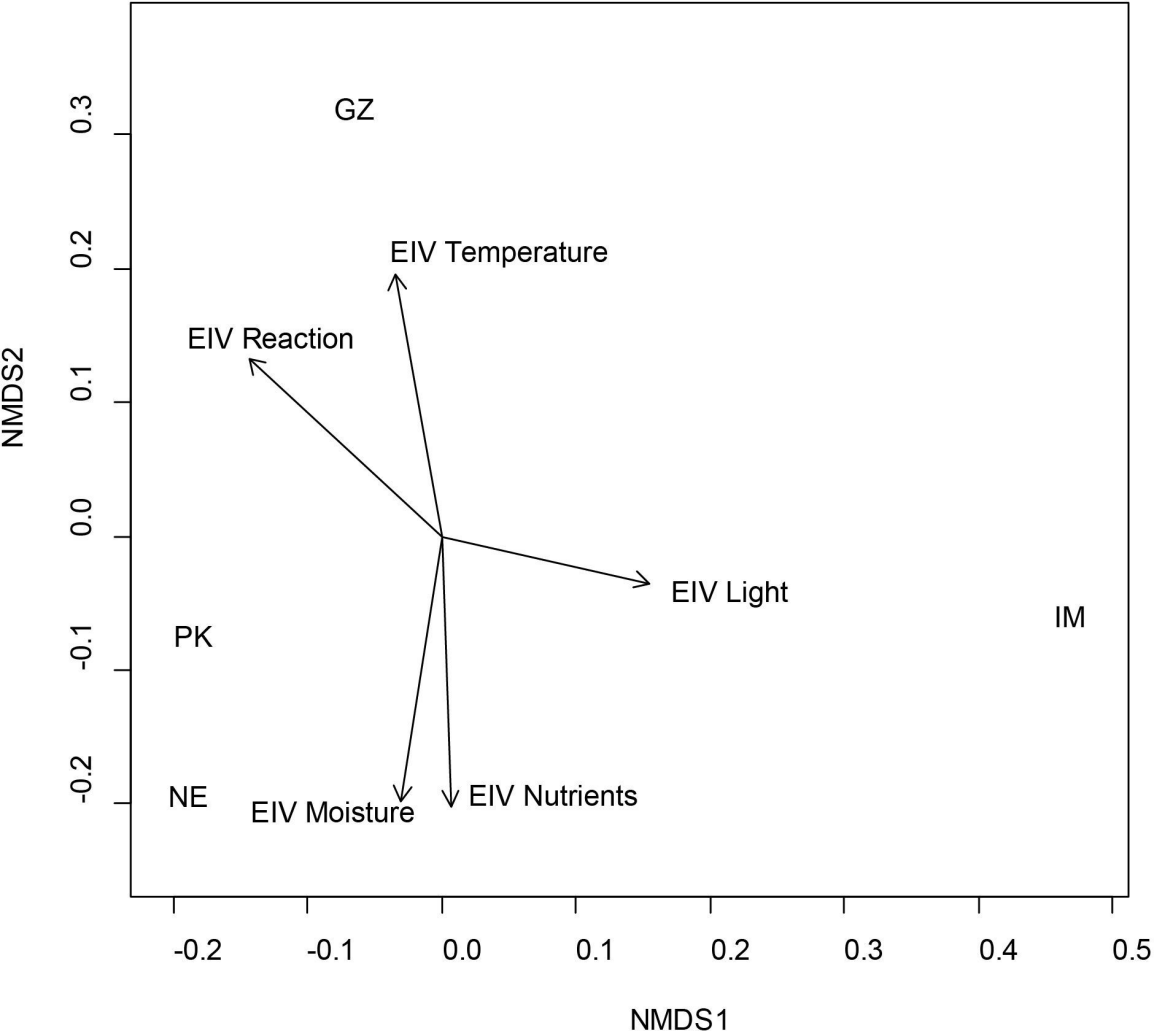
NMDS ordination diagram with passively projected Ellenberg indicator values. EIV are passively projected as vectors plotted on the bi-dimensional space. Five EIV are included: Light, Temperature, Moisture, Reaction and Nutrients (shown in black arrows). Locations: PK–Paklenica; NE–Neretva; IM–Imotski; GZ—Gizdavac.

The NMDS ordination diagram (Fig 2) reveals the relationships between the distinguished woody plant species in the locations in terms of the main ecological gradients, described by Ellenberg indicator values. In the Imotski location, the abundance of more heliophile (i.e., light-demanding) species was certainly increased. In the Gizdavac location, woody plant species were separate according to temperature and soil reaction and in the Paklenica and Neretva locations according to moisture and soil nutrient availability. Paklenica wild grapevine population grows in the coldest and humid habitat according to climatic variables (CV) (Table 1) and Ellenberg indicator value (EIV) (Fig 2) while Gizdavac population grows in the driest and the warmest habitat according to CV and EIV (Table 1, Fig 2). Wild grapevine population in Neretva grows in the wettest habitat according to average annual precipitation (AP) and EIV (Table Table 1, Fig 2). For temperature the opposite trend was observed in this location–warmest habitat according to average annual temperature (AT) and coldest according EIV (Table Table 1, Fig 2).

The differences between Ellenberg indicator values in the locations were small and statistically insignificant. The average values for light and temperature reflect the light-demanding character of most of the analyzed species and arid Mediterranean-mountainous environment conditions. The average moisture, soil reaction values and soil nutrient content indicate low soil humidity and mildly acidic, nutrient-poor soils (Table 5).

**Table 5:** Mean Ellenberg indicator values of the four locations.

Location	Mean Ellenberg indicator value				
	Light	Temperature	Moisture	Soil reaction	Soil nutrient
**Paklenica**	6.3	6.6	3.6	6.6	4.0
**Neretva**	5.7	6.9	3.9	5.9	4.1
**Imotski**	6.9	7.1	3.5	5.1	4.0
**Gizdavac**	6.3	7.5	3.1	6.6	3.7

## Discussion

Only recently, wild grapevines encompassing a large number of individuals was phenotypically described and genetically fingerprinted in the Balkan Peninsula in Croatia and Bosnia and Herzegovina [9]. In this study of 53 wild grapevine individuals, the allelic variation at eight polymorphic cpSSR loci was determined. In addition, for the first time, the woody species community that is part of the wild grapevine habitat was analyzed in an Eastern Adriatic area.

According to the assessed environmental factors, the ecosystems in which wild grapevines were found in our study were forest ecosystems in the contact zone of the EU-Mediterranean and the sub-Mediterranean. Characterized by a typical karst relief of calcocambisol soil, an average temperature of 11°C and precipitation of 950mm, the grapevine locations can be characterized as arid Mediterranean with a temperate climate and poor soil. To express the wild grapevines’ ecological affinities and environmental preferences we used Ellenberg's indicator value to each associated woody species from the wild grape habitat. Mean Elenberg indicator value and NMDS (Table 5, Fig 2,) highlights oligotrophic, termophilous, xerophilous, semi shade character of the species. The opposite trend in species separation between climate data and EIV according the temperature was observed in Neretva location (Table 4, Fig 2). This difference is due to the fact that the average annual temperature values were measured at the nearest meteorological station, while the EIV reflected the microclimate at a specific habitat.

As expected, the distribution and incidence of the accompanying woody species was not homogenous and differed in relation to the preservation stage of the location and to a minor extent in type and depth of terrain and water fluctuation. The two best-preserved locations harbored the largest biodiversity in plant woody species: Neretva, 39; Paklenica, 31 (Table 4). These populations appear ecologically quite similar. The ecological affinity between these two wild grapevine population highlighted by either EIV NMDS (Fig 2) show similar positions and trends. Composition of vegetation and presence of climax species (*Quercus ilex*, *Q*. *pubescens*, *Pinus nigra*, *P*. *halepensis*) show that forest have reached a steady state. As a consequence of the best preserved forests in these locations the largest number of wild grapevine individuals was also identified: Neretva, 20; Paklenica, 19 (Table 4).

In contrast, the Imotski population was found growing on rocky terrain in very shallow and scarce soil such that the associated woody plants were characterized by rocky vegetation and poor flora (Table 4). As a consequence, only 8 woody plant species were found. In this location, wild grapevines crawled along the rocky ground, instead of climbing as usual. Because the water level in the lake varies with the season, with the peak in the winter, the populations are very often completely under water. Although in this location wild grapevine population grows on scarcely evolved soils, subjected to a certain degree of erosion, presence of 11 individuals in a small area indicate that it is well adapted on specific edaphic conditions.

In Gizdavac location sixteen supporter plants were found and only three wild grapevine individuals were identified. Due to grazing, forest management activities by local inhabitants through the past, structure and composition of vegetation were changed. Woody species are in a shrub form what is not known in undisturbed natural formations as in the gallery forests.

However, according to the list of species and their occurrance (Table 4. Fig 1) the following considerations can be made: the wild grapevine in the Eastern Adriatic region lives often associated with: *Rubus sp*. and also common with other lianae as: *Clematis vitalba* and *Hedera helix*. On three of the four locations, the presence of *Fraxinus ornus* and *Carpinus orientalis* was indicative. *Prunus mahaleb* and *Ficus carica* represented the community closest to wild grapevines. These species functioned as physical supporter plants for grapevine growth, and grapevines were found forming climbing lianas on these species. The components of the climax vegetation in the wild grapevine habitat were in accordance with those detected a short distance from a watercourse in Andalusia, Spain [34]. As a result of the climatology and edaphic conditions of our locations, no floodplain populations were found, in contrast to wild grapevine habitats in western Spain [35] and Central European countries [4].

As with woody species diversity, the haplotype distribution based on cpDNA was also not homogenous in our study (Table 3). The dominance of haplotype A (or group IV) in three of four locations with a frequency of more than 50% places our results in the trend found for other wild grapevine accessions across Europe [36–37], which find haplotype A as the most frequently distributed in European wild accessions. In our study, the greater incidence of haplotype A was found in the best-preserved locations, Neretva and Paklenica, where the larger variability in plant woody species was also found. In contrast, haplotype D, which corresponds to group I, was the most frequent haplotype in the Imotski population. According to De Mattia et al. [37] and Arroyo-Garcia et al. [36], this haplotype is the most frequent in domesticated grapes and completely absent in the wild pool of central and western Europe (France, Spain, Portugal, Austria and Germany), whereas it is present in *V*. *Vinifera* ssp. *sylvestris* in the Italian Peninsula. The 80% frequency of haplotype D found in the Imotski wild grapevine population in combination with dominant haplotype A found in the other tested populations makes this area unique in terms of haplotype combinations.

Not only haplotype richness or type but also the combination of haplotypes must be considered when describing grapevine history. According to certain authors, haplotype distribution can be associated with specific geographic areas [23, 38]. For instance, the greatest haplotype diversity (seven chlorotypes) was found in the wild grape populations of the Near East and Caucasus, which is the presumed primary center of domestication. Chlorotypes D, B and C are present in Turkish wild accessions [39]. In both studies, the prevalent haplotype was D, which is not common in the majority of European wild populations. Is it possible that wild grapevines from this part of the Balkan Peninsula have conserved chlorotype D from ancient times when wild grapevines were colonizing Europe from the East. Moderate and low chlorotype diversity is found in Western Europe. In the Iberian Peninsula, haplotypes A and B (IV and II) were found [17, 38], whereas in France, Germany and Austria, haplotype A (IV) was recorded among wild germplasms. In the Italian Peninsula, haplotypes A and D (IV and I) were found [38]. The same year, Grassi reported the presence of three more chlorotypes in addition to A and D in Italian wild grape populations [20].

The present state of haplotype diversity in wild grapevines is a result of the genetic inheritance, environmental factors, civilization and spread of diseases that formed the present state of grapevine distribution and shaped the observed distribution of global plant biodiversity and therefore wild grapevines [24]. It is believed that during the Quaternary glaciations, wild grapevines were restricted to the southern parts of the Iberian, Italian and Caucasus regions [40]. Croatia and Bosnia and Herzegovina are located between these areas in the Eastern Adriatic Region with a suitable climate and sufficient natural habitat for wild grapevines to be found and investigated. The presence of plentiful underground water and the dynamic karstic relief observed in this study could have enabled the formation of a microclimatic area suitable for grapevine refuges during unfavorable climatic conditions. In contrast to the southern richness in biodiversity, northern areas were found to be inhabited by rare wild populations, essentially consisting of single haplotypes [20] because unfavorable climatic conditions had impoverished plant biodiversity.

If the Italian peninsula was a hotspot for the secondary colonization of wild grapevines that occurred during postglacial recolonization [40], as has been described for *Fagus sylvatica*, *Castanea sativa*, *Quercus robur*, *Q petrea* and *Q pubescens*, it is possible to hypothesize that the Eastern Adriatic had a role as a refuge for wild grapevines during the last glaciation. That Croatia harbors and shares haplotypes common in the Italian wild germplasm indicates a connection between these two countries in the past. In our study, *Fagus sylvatica* was found at the Paklenica site. The prevalence of *Q*. *pubescens* at the Neretva site could also support this theory. Grassi et al. [20] identified *Q*. *pubescens* among species that survived the last glaciation. During the last glacial maximum, the level of the Adriatic Sea was low [41], leading to the emergence of lowlands and contacts between the Italian and Balkan floras and enabling the easy migration of plants and gene flow between the two countries. The rivers of the Croatian and Italian mainland were in close contact because the Neretva, Cetina and Po rivers were longer than today and their mouths extended deeper into the Adriatic Sea [41]. It is not surprising for this surviving species to be found in Croatia, Bosnia and Herzegovina and Italy with the same haplotype as in wild grapevines.

Out results shows that different stage of area protection has an impact on biodiversity and number of wild grapevine individuals. During the period of time, human impact, grazing, wildfire, competition of other plants: lianas (*Hedera helix* and *Clematis vitalba*), invasive species (*Ailanthus altissima*) are the major hazard to be controlled. Vertical structure of vegetation and presence of water will also de essential to provide a sustainable environment. These wild grapevine populations constitutes a rare endangered plant in the Eastern Adriatic region, ant it is necessary to start activities addressed to its in situ and ex situ protection. The most efficient way to conserve endangered plant species is to protect their natural habitats and ecosystems.

## Conclusion

The haplotype diversity of wild grapevines in the Eastern Adriatic region and the diversity of the associated woody plant species were presented. The presence and frequency of chloroplast haplotypes A and D in *V*. *vinifera* ssp. *sylvestris* habitats on Croatian and Bosnian and Herzegovinian territory and on the Italian Peninsula lead us to conclude that these areas were once connected and shelter areas for wild grapevines. Filling knowledge gaps regarding the chloroplast genetic diversity of present-day wild grapevines but also of cultivated grapevines, and their relationship with domesticated grapes can contribute to a better understanding of grapevine domestication and its evolution in general. Investigating wild grapevines, we investigate history and processes that occurred millennia ago. However, the advanced fragmentation and erosion of wild genetic pools should be prevented for further research on wild grapevines to remain plausible.
